# Supplementary material for: PYK2 senses calcium through a disordered dimerization and calmodulin-binding element
Source: Commun Biol. 2022 Aug 9;5:800. doi: 10.1038/s42003-022-03760-8 (PMC9363500; doi:10.1038/s42003-022-03760-8)
Supplement: Supplementary file 2 — Supplementary Information [file 42003_2022_3760_MOESM2_ESM.pdf]

Supplementary Figures

PYK2 senses calcium through a disordered dimerization and calmodulin-binding element

Afaque A. Momin[1], Tiago Mendes[2#], Philippe Barthe[3#], Camille Faure[2], SeungBeom Hong[1], Piao Yu[1], Gress Kadaré[2], Mariusz Jaremko[4], Jean-Antoine Girault[2], Łukasz Jaremko[4], Stefan T. Arold[*1,3]

[1]King Abdullah University of Science and Technology (KAUST), Computational Bioscience Research Center (CBRC), Division of Biological and Environmental Science and Engineering (BESE), Thuwal, 23955-6900, Saudi Arabia

[2]Inserm UMR-S 1270, Sorbonne Université, Faculty of Sciences and Engineering, Institut du Fer à Moulin. Paris, 75005, France

[3]Centre de Biologie Structurale (CBS), University Montpellier, INSERM U1054, CNRS UMR 5048, F-34090, Montpellier, France

[4]King Abdullah University of Science and Technology (KAUST), Division of Biological and Environmental Science and Engineering (BESE), Thuwal, 23955-6900, Saudi Arabia

[#]contributed equally

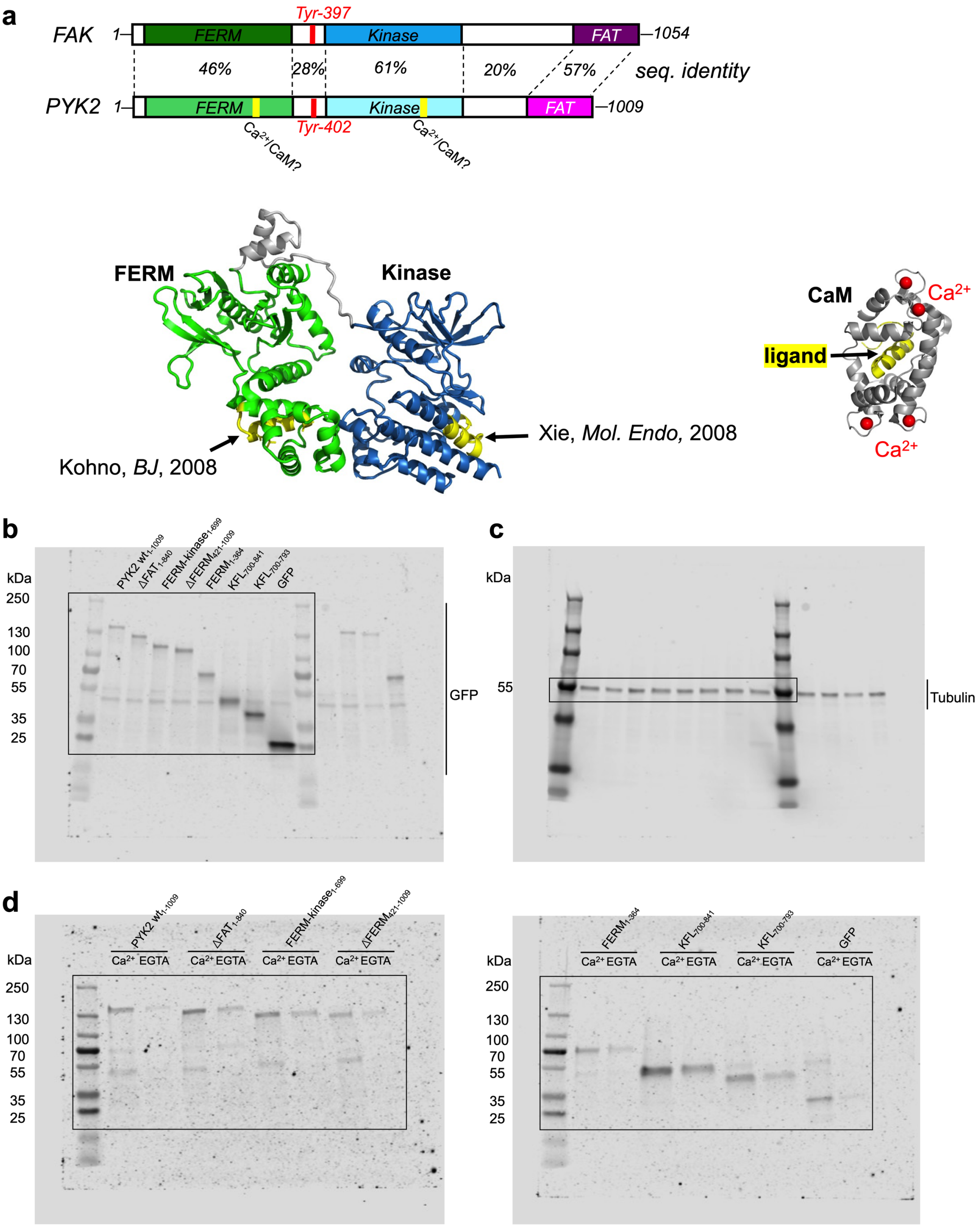

Supplementary Figure 1: (a) Proposed non-KFL–specific CaM interactions. (a) Top: Schematic overview of FAK and PYK2, showing their domain structure and sequence identity. The localization of the previously proposed Ca^{2+}/CaM binding motifs is indicated in yellow. Bottom, left: Position of the previously proposed Ca^{2+}/CaM binding motifs (coloured in yellow) within the three-dimensional structures of the FERM domain (green, as proposed by Kohno et al.[1]) and kinase domain (blue; as proposed by Xie et al.[2]). Bottom, right: In these locations, the proposed Ca^{2+}/CaM binding motifs would not be able to associate with CaM in a canonical way, where calcium (red)-bound CaM (gray) wraps around the helical ligand (yellow). Uncropped images of the representative immunoblot of input fractions of the indicated GFP-tagged PYK2 constructs immunolabeled with **(b)** GFP and **(c)** tubulin antibodies as shown in Fig. 1b. **(d)** Uncropped images of representative GFP immunoblot of GFP-PYK2 constructs associated with the CaM Sepharose beads as shown in Fig 1c. Boxed areas represent the cropped part of the immunoblots.

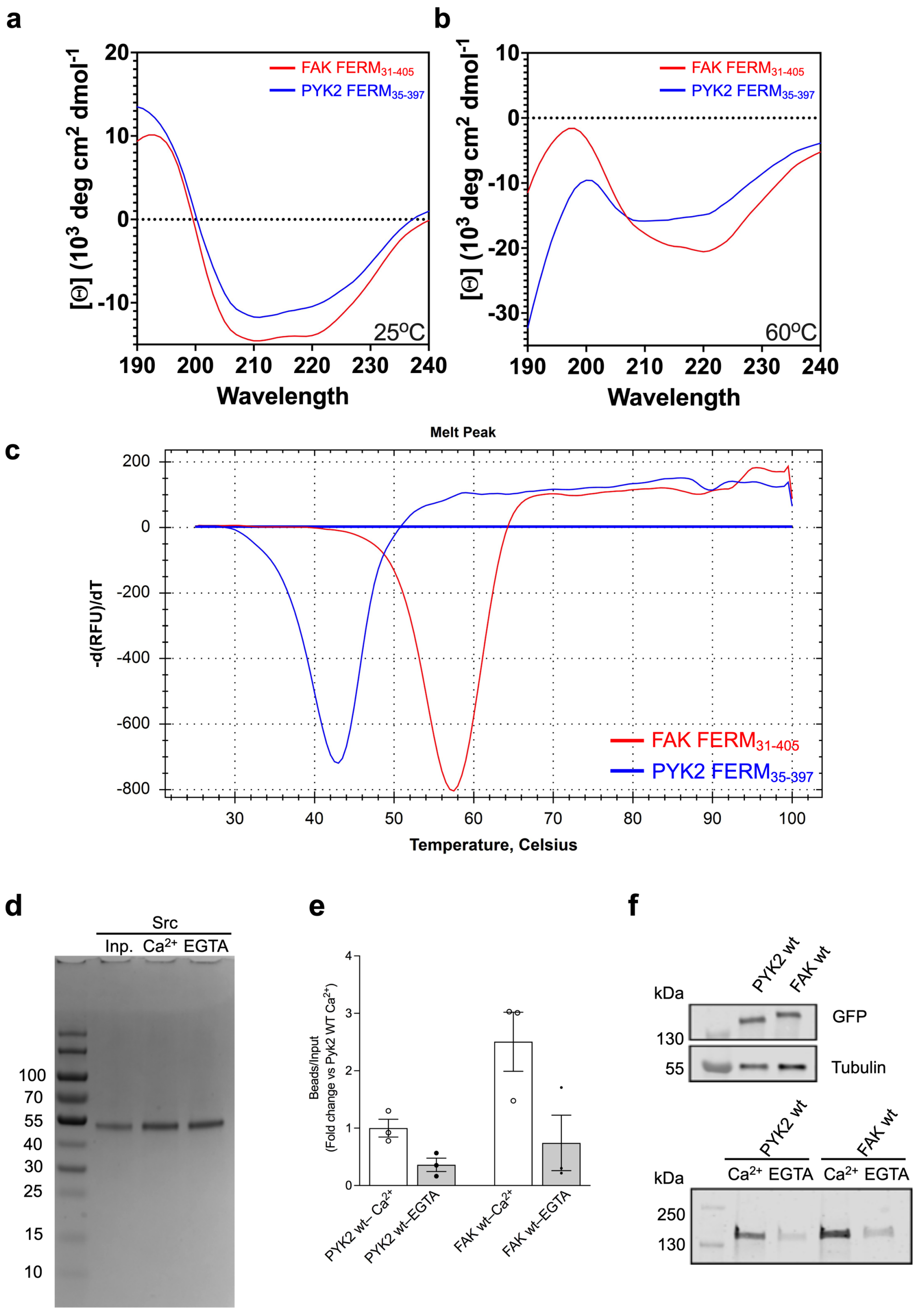

Supplementary Figure 2. CaM agarose bead assays and associated experiments. Circular Dichroism (CD) spectra for FAK $FERM_{31-405}$ and PYK2 $FERM_{35-397}$ at **(a)** 25 °C and **(b)** 60 °C. **(c)** Differential scanning fluorimetry (DSF) curve for FAK $FERM_{31-405}$ (melting temperature, *Tm* = 57.8 °C) and PYK2 $FERM_{35-397}$ (*Tm* = 43.4 °C). **(d)** CaM agarose bead assay using recombinant and purified Src. Bands correspond to Src retained by the CaM beads in the presence (Ca^{2+}) or absence (EGTA) of Ca^{2+}. Inp.: input. The *Mw* of the markers are given in kDa. **(e)** CaM agarose bead assay using cell lysates. Graphical representation of GFP densitometry in beads normalized by input, presented as fold change with respect to PYK2 WT Ca^{2+} bead fraction. Bars correspond to the mean of 3 independent experiments, ± SEM. **(f)** *Top*: Representative image of input fractions immunolabeled for GFP tagged full-length PYK2 and FAK constructs and tubulin. *Bottom*: Representative image of bead fractions immunolabelled for full-length PYK2 and FAK. Binding experiments are represented as (mean ± SD, n=3).

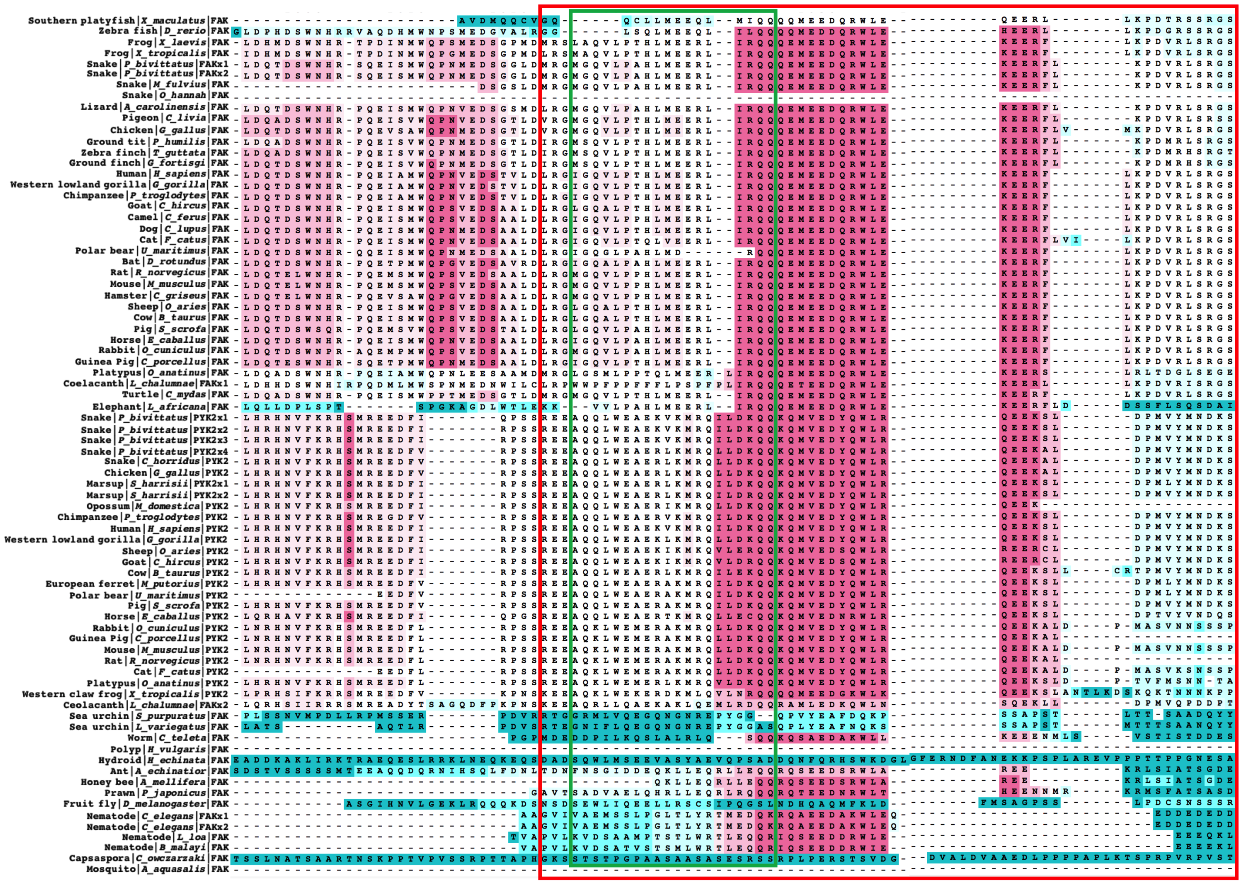

Supplementary Figure 3. Sequence conservation within the PYK2 and FAK KFL. Sequence alignment for 78 FAK or PYK2 sequences from different species. Sequence conservation is colour-ramped from dark cyan (divergent) to dark pink (highly conserved). Red box indicates helical region found bioinformatically, while the green box indicates the experimentally known helical region by NMR.

a

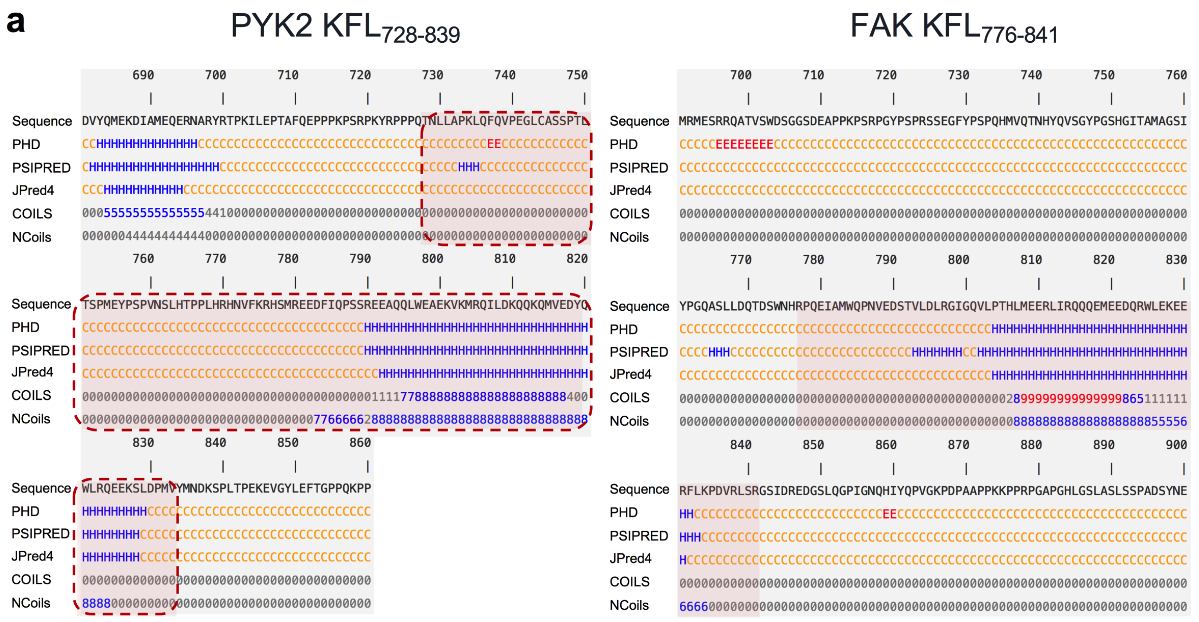

b

CaM binding patterns - literature

CaM binding motifs	Sequence
1–10	[FILVW]xxxxxxxx[FILVW]
1–5-10	[FILVW]xxx[FAILVW]xxxx[FILVW]
Basic 1–5–10	[RK][RK][RK][FAILVW]xxx[FILV]xxxx[FILVW]
1–12	[FILVW]xxxxxxxxxx[FILVW]
1–14	[FILVW]xxxxxxxxxxxx[FILVW]
1–8–14	[FILVW]xxxxxx[FAILVW]xxxxx[FILVW]
1–5–8–14	[FILVW]xxx[FAILVW]xx[FAILVW]xxxxx[FILVW]
Basic 1–8–14	[RK][RK][RK][FILVW]xxxxxx[FAILVW]xxxxx[FILVW]
1–16	[FILVW]xxxxxxxxxxxxxx[FILVW]
IQ	[FILV]Qxxx[RK]Gxxx[RK]xx[FILVWY]
IQ-like	[FILV]Qxxx[RK]xxxxxxxx
IQ-2A	[IVL]QxxxRxxxx[VL][KR]xW
IQ-2B	[IL]QxxCxxxxKxRxW
IQ unconventional	[IVL]QxxxRxxxx[RK]xx[FILVWY]

Ca²⁺ independent

CaM binding motifs - KFL of PYK2

Motif	Sequence	Residues
1–10	LLAPKLQFQV	730-739
1–10	ILDKQQKQMV	807-816
1-12	VPEGLCASSPTL	739-750
1–12	LHTPPLHRHNVF	763-774
1–12	VFKRHSMREEDF	773-784
1–12	FKRHSMREEDFI	774-785
1–12	IQPSSREEAQQL	785-795
1–12	LWEAEKVKMRQI	796-807
1–12	WEAEKVKMRQIL	797-808
1–12	LRQEEKSLDPMV	822-833
1-14	LLAPKLQFQVPEGL	730-743
1-14	FQVPEGLCASSPTL	737-750
1-14	LTSPMEYPSPVNSL	750-763
1-14	VNSLHTPPLHRHNV	760-773
1-14	FIQPSSREEAQQLW	784-797
1-14	LDKQQKQMVEDYQW	808-821
1-14	VEDYQWLRQEEKSL	816-829
1-16	LQFQVPEGLCASSPTL	735-750
1-16	ILDKQQKQMVEDYQWL	807-822
1-8-14	LLAPKLQFQVPEGL	730-743
IQ-LIKE	IQPSSREEAQQLWE	785-799

Supplementary Figure 4. Bioinformatic analysis of the PYK2 and FAK KFL. (a) Secondary structure and coiled-coil prediction using multiple servers for the KFL region in PYK2 (left) and FAK (right). Programs used (PHD[3], PSIPRED[4], JPred4[5], COILS[6] and Waggawaga webserver for NCOILS[7]. PYK2 $KFL_{728\text{-}839}$ and FAK $KFL_{776\text{-}841}$ are shaded with salmon color, while the PYK2 region which was predicted by the Calmodulation database and meta-analysis predictor (cam.umassmed.edu) to contain CaM binding sites is outlined with red dotted lines. **(b)** *Left*: Established CaM binding motifs, obtained from cam.umassmed.edu, and inspired by [8]. The numbers in the CaM binding motifs column indicate the positions in the motif that require a hydrophobic residue. In the Sequence column, residues in brackets comply with the consensus, and x indicates any residue. *Right*: CaM binding motifs identified in the KFL region of PYK2.

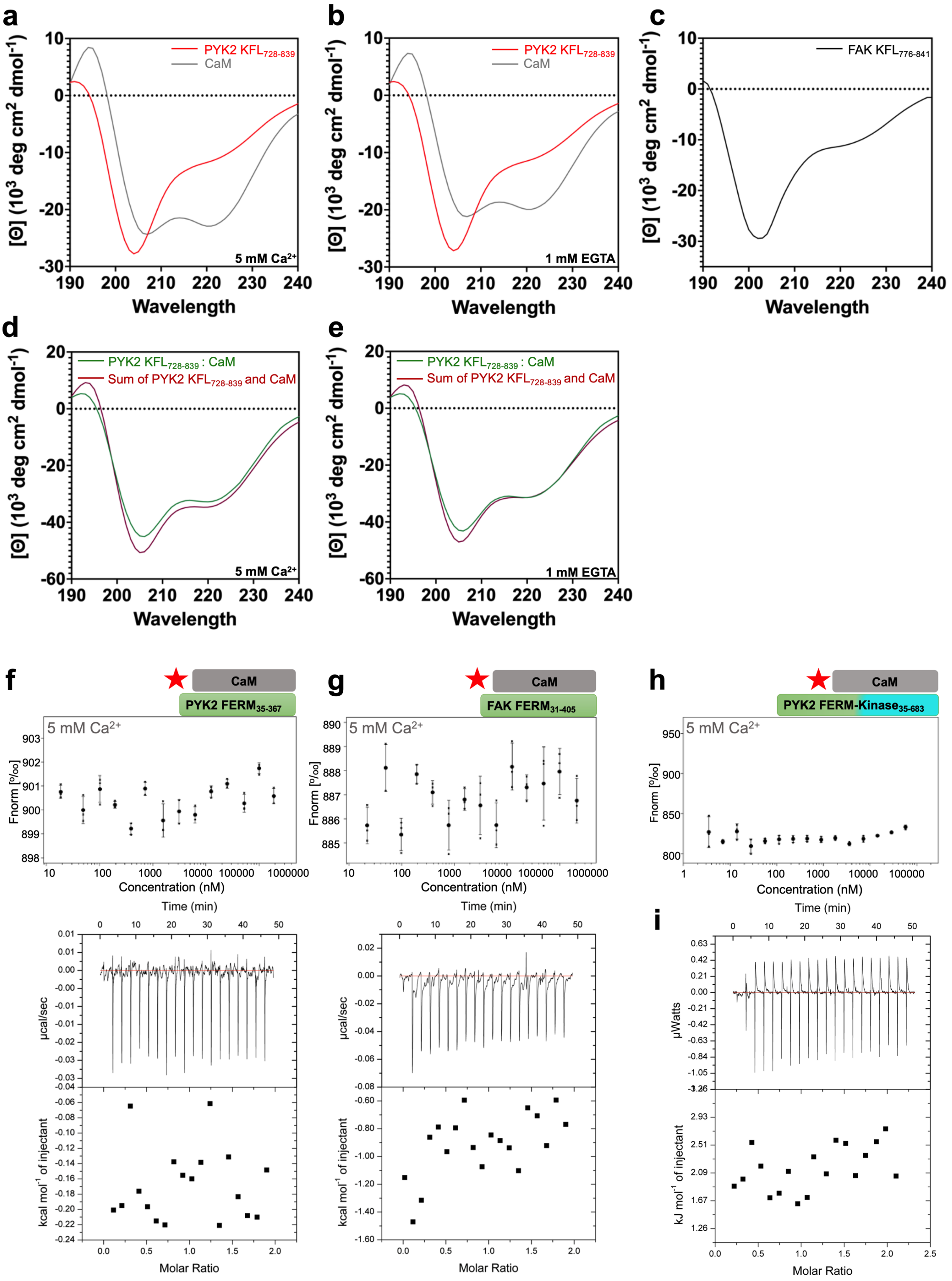

Supplementary Figure 5. Biophysical analysis of PYK2 and FAK KFL. CD spectra of PYK2 KFL$_{728-839}$ and CaM in the presence **(a)**, and in the absence **(b)** of Ca^{2+}. **(c)** CD spectrum of FAK KFL$_{776-841}$. CD spectra of PYK2 KFL$_{728-839}$: CaM complex in the presence **(d)** and absence **(e)** of Ca^{2+} overlapped on the sum of the individual spectra of PYK2 KFL$_{728-839}$ and CaM shown in **(a)** and **(b).** MST and ITC experiments probing the association of Ca^{2+}/CaM with FERM domains of **(f)** PYK2 and **(g)** FAK. **(h)** MST experiment testing binding of the PYK2 FERM-kinase fragment with Ca^{2+}/CaM. The red star indicates the fluorescently labelled protein. **(i)** ITC experiment probing the interaction of PYK2 KFL$_{728-839}$ with calcium. Binding experiments are represented as (mean ± SD, n=3).

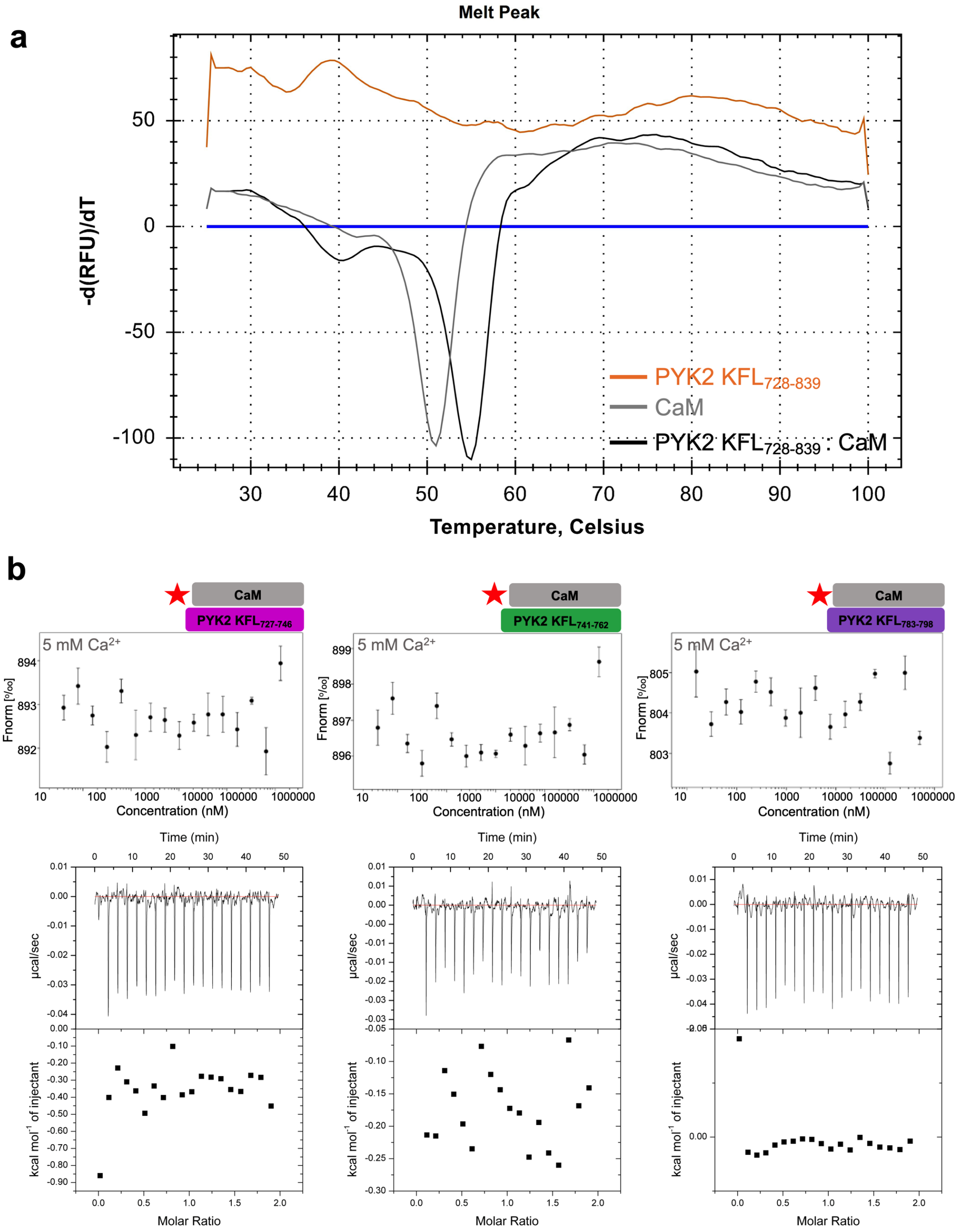

Name	Sequence	*Mw* (Da)
PYK2 $KFL_{727-746}$	QTNLLAPKLQFQVPEGLCAS	2157
PYK2 $KFL_{741-762}$	EGLCASSPTLTSPMEYPSPVNS	2267
PYK2 $KFL_{783-798}$	DFIQPSSREEAQQLWE	1968

Supplementary Figure 6. Biophysical analysis of PYK2 KFL and CaM. (a) Differential scanning fluorimetry (DSF) curve, recorded in the absence of Ca^{2+}, for PYK2 $KFL_{728-839}$ alone (orange), CaM alone (gray) and CaM in the presence of PYK2 $KFL_{728-839}$. **(b)** Probing the capability of PYK2 KFL–derived peptide motifs to associated with CaM. *Top*: MST and ITC binding curves testing binding of synthesized peptides covering the bioinformatically identified CaM binding motifs within PYK2 $KFL_{728-839}$. Red star indicates labelled protein. *Bottom*: Sequence and molecular weight of the peptides used. Binding experiments are represented as (mean ± SD, n=3).

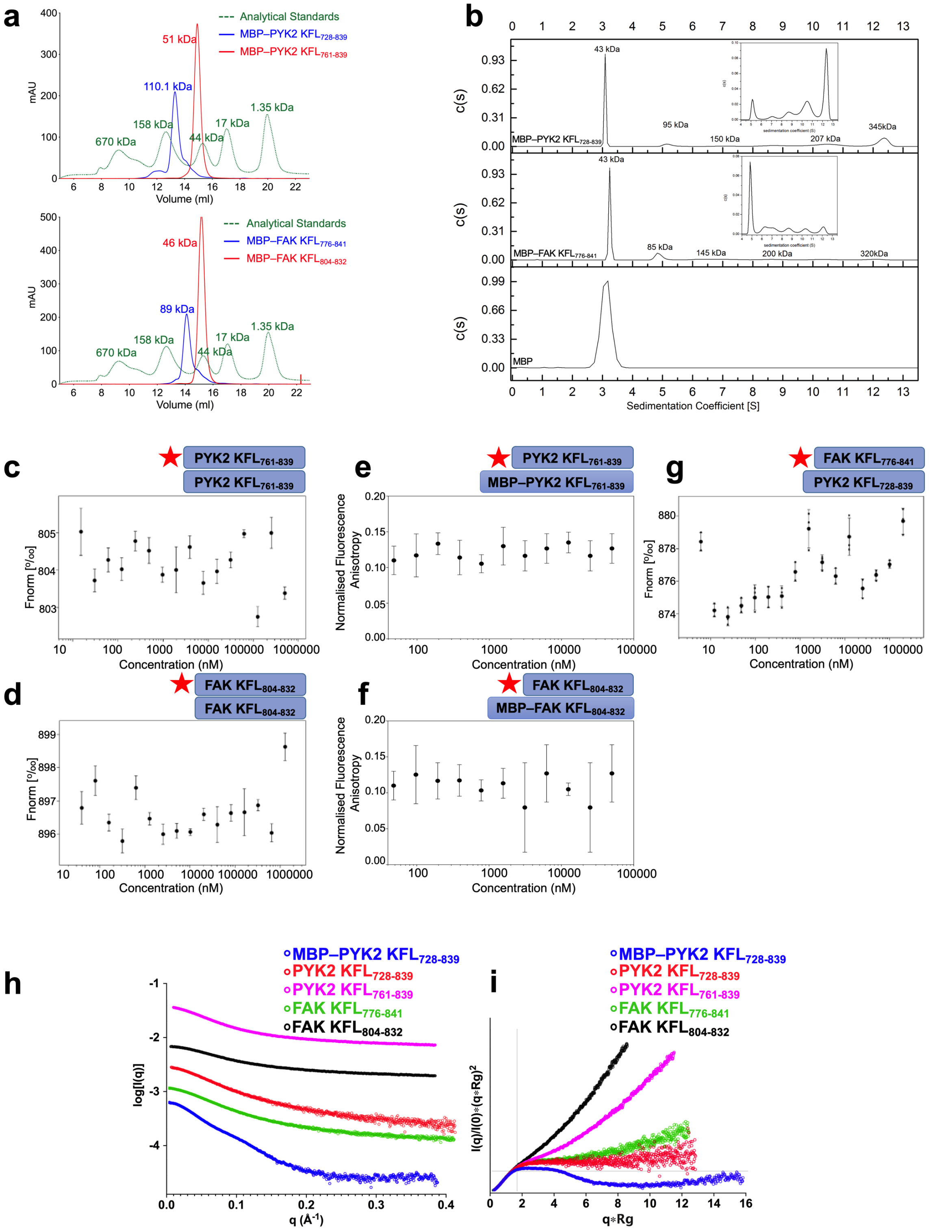

Supplementary Figure 7: Biophysical assays testing the dimerization and structural features of KFL fragments from PYK2 and FAK. (a) SEC elution profiles. Mw of KFL constructs and of the analytical standards are given. **(b)** Analysis of AUC sedimentation velocity data. **(c,d)** MST and **(e,f)** fluorescence anisotropy studies of KFL fragments that fail to show dimerization. **(g)** MST study between PYK2 $KFL_{728\text{-}839}$ and FAK $KFL_{776\text{-}841}$ that fail to show interaction. The red star indicates the labelled protein. **(h)** SEC–SAXS profiles and **(i)** KRATKY plots derived from **(h)**. Binding experiments are represented as (mean ± SD, n=3).

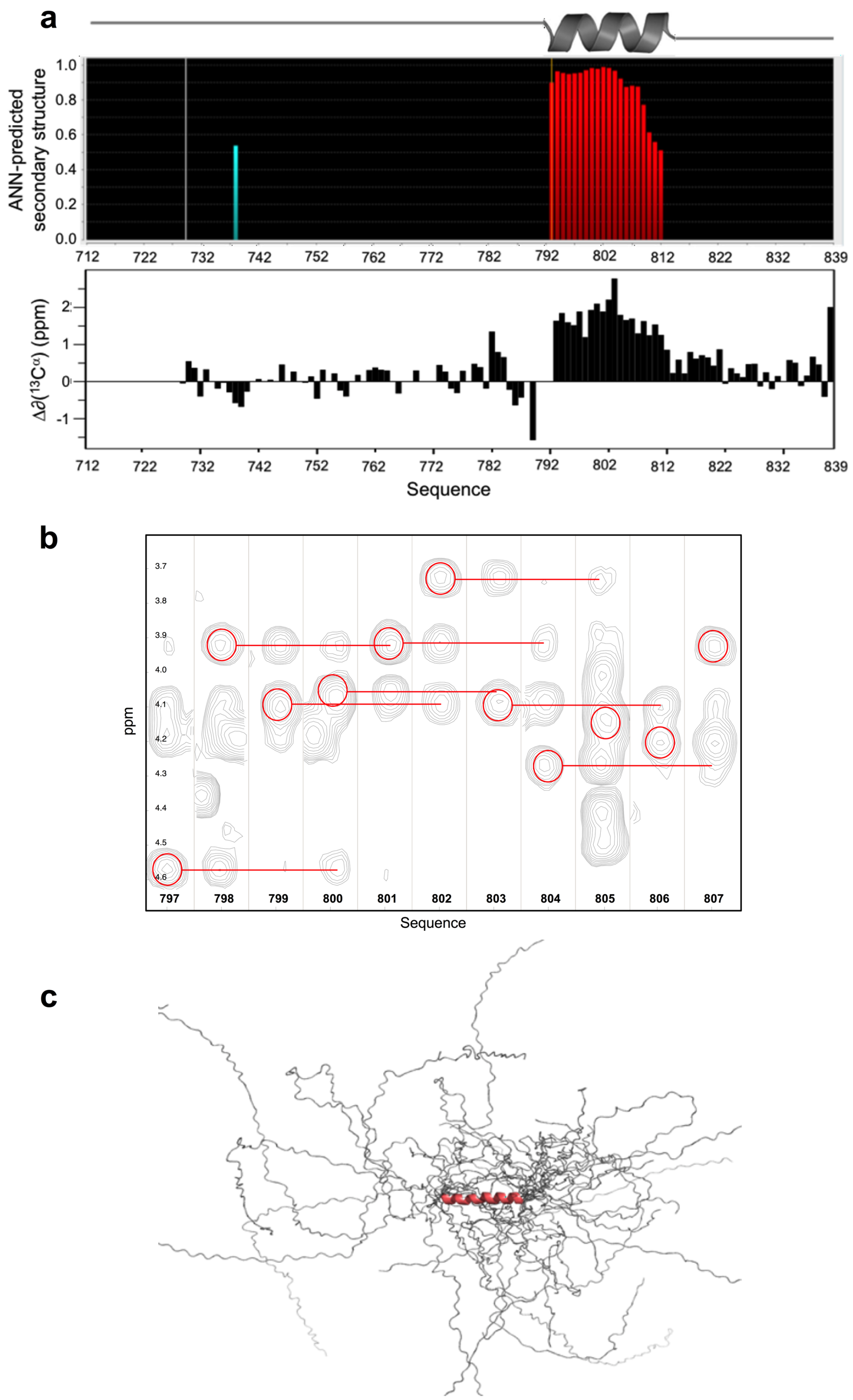

Supplementary Figure 8. NMR analysis of PYK2 KFL$_{728\text{-}839}$. (a) Secondary structure derived from Artificial Neural Network (ANN) prediction by TALOS-N (https://spin.niddk.nih.gov/bax/software/TALOS-N/;[9] using the backbone ($^{13}C^{\alpha}$, $^{13}C'$, ^{15}N, $^{1}H^{\alpha}$ and $^{1}H^{N}$) and $^{13}C^{\beta}$ chemical shift assignments. **(b)** Extract from NOESY (150 ms) strip plot. The strips are ordered according to the sequence. The contacts *i*, *i+3* of the Hα (red circle) with the HN are shown by red lines. **(c)** 3D structures of the PYK2 KFL$_{728\text{-}839}$. The helical region is shown in pink.

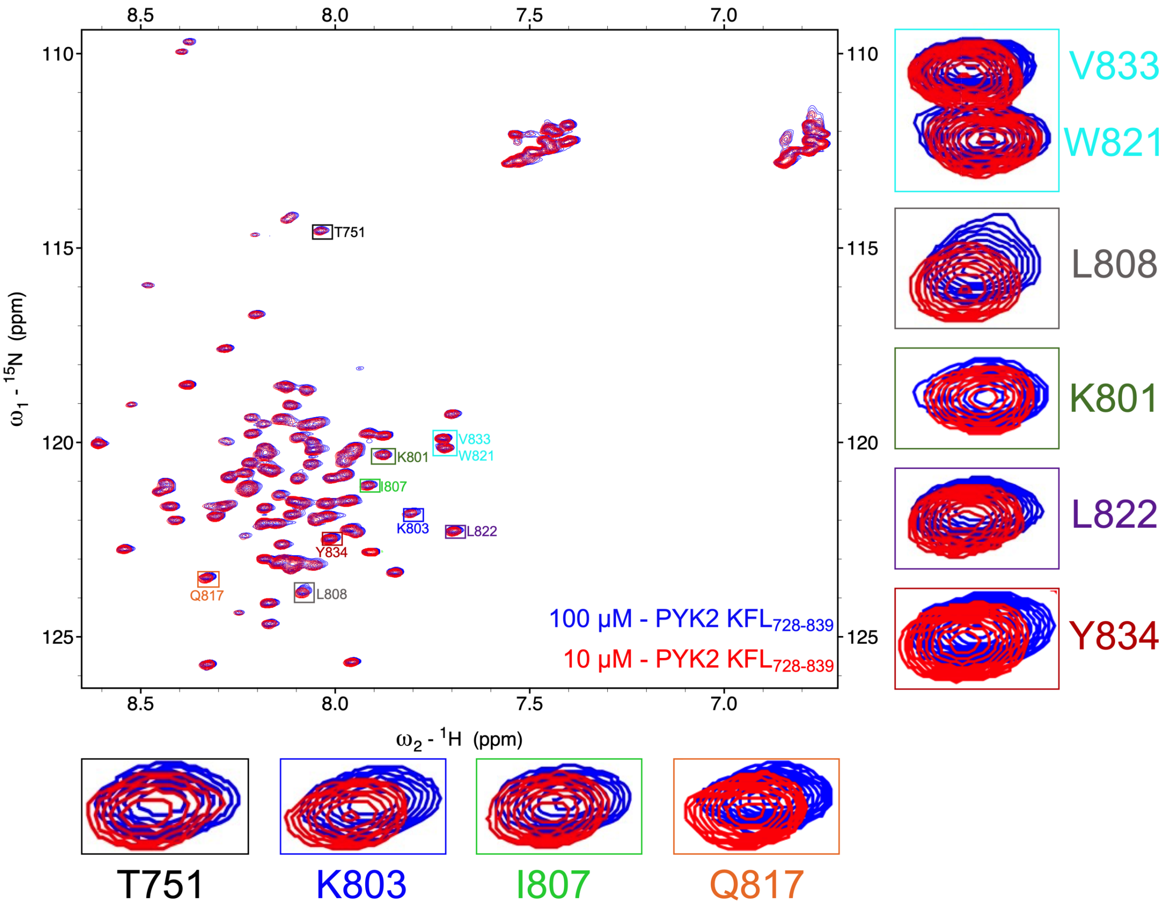

Supplementary Figure 9. [^{1}H,^{15}N] HSQC overlay for [^{13}C,^{15}N] PYK2 KFL$_{728\text{-}839}$ at 100 µM (blue) and 10 µM (red) to analyse CSPs upon changes in the monomer:dimer ratio. To support that CSPs were not simply a general result of small changes in temperature or pH, we also displayed two peaks that did not move upon dilution (K801 and W821).

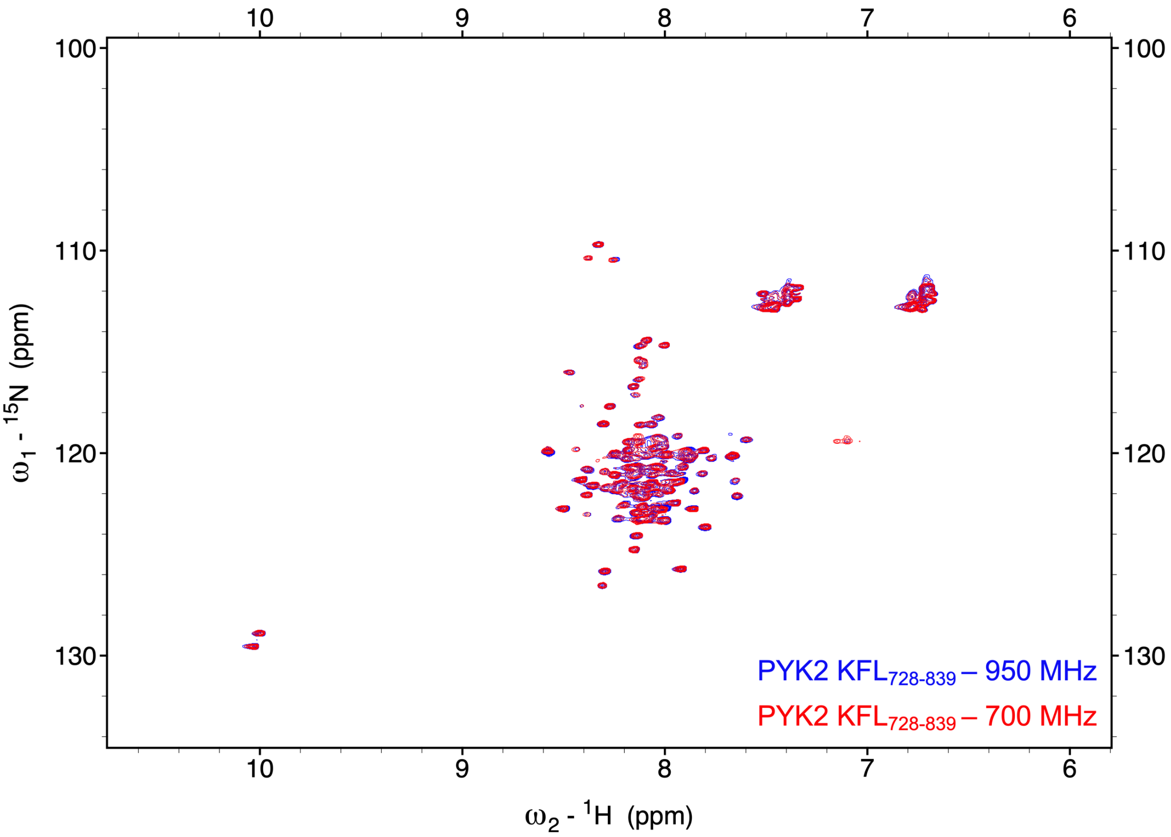

Supplementary Figure 10. [^{1}H,^{15}N] HSQC overlay for [^{13}C,^{15}N] PYK2 KFL$_{728\text{-}839}$ at 100 µM concentration measured at 950 MHz (blue) and 700 MHz (red).

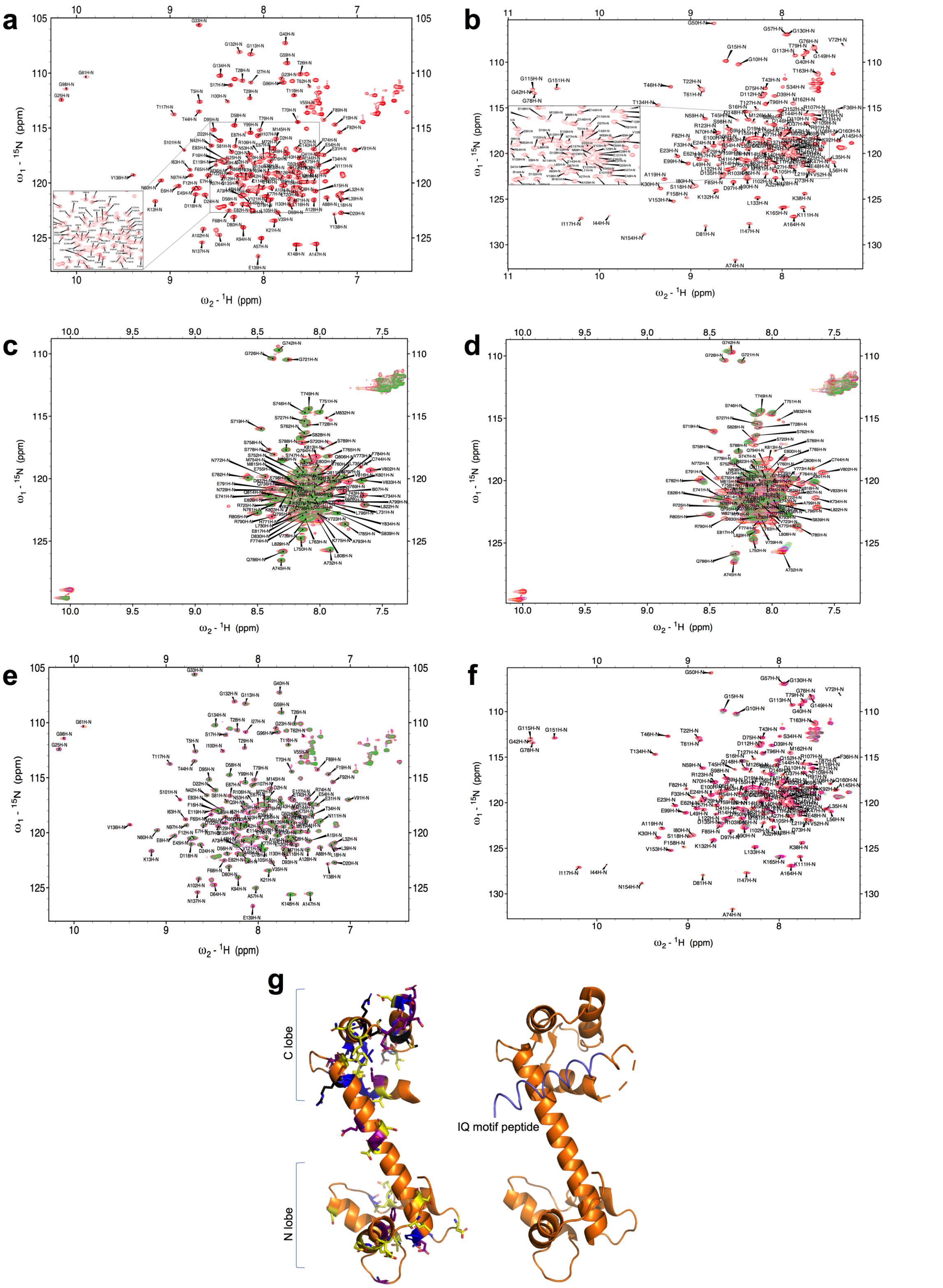

Supplementary Figure 11. NMR analysis of CaM–PYK2 KFL interactions. [^{1}H,^{15}N] HSQC spectra for the NMR backbone assignment of **(a)** apo-CaM and **(b)** Ca^{2+}/CaM **(c)** [^{1}H,^{15}N] HSQC overlay for PYK2 KFL$_{728\text{-}839}$ in the absence (red) and presence of 0.5 (orange), 1 (magenta), 2 (blue), 4 (beige), and 8 (green) times molar excess of CaM in buffer containing 1 mM EGTA. **(d)** As (c) but in the presence of 5 mM Ca^{2+}. **(e)** [^{1}H,^{15}N] HSQC overlay for apo-CaM in the absence (red) and presence of 0.5 (orange), 1 (magenta), 2 (blue), 4 (beige), and 8 (green) times molar excess of PYK2 KFL$_{728\text{-}839}$ in buffer containing 1

mM EGTA. **(f)** As **(e)**, but in the presence of 5 mM Ca^{2+}. **(g)** *Left*: The CSP data for [^{13}C,^{15}N] apo-CaM obtained during the titration with PYK2 $KFL_{728-839}$ was mapped on the 3D structure of apo-CaM (PDB ID: 4e53). Residues that showed major shifts are coloured purple, minor shifts coloured yellow and residues for which resonances disappeared are coloured blue. Unassigned and prolines coloured black. *Right*: The 3D structure of the neuromodulin IQ peptide (blue) bound to apo-CaM (gold; PDB ID: 4e53) is shown for comparison.

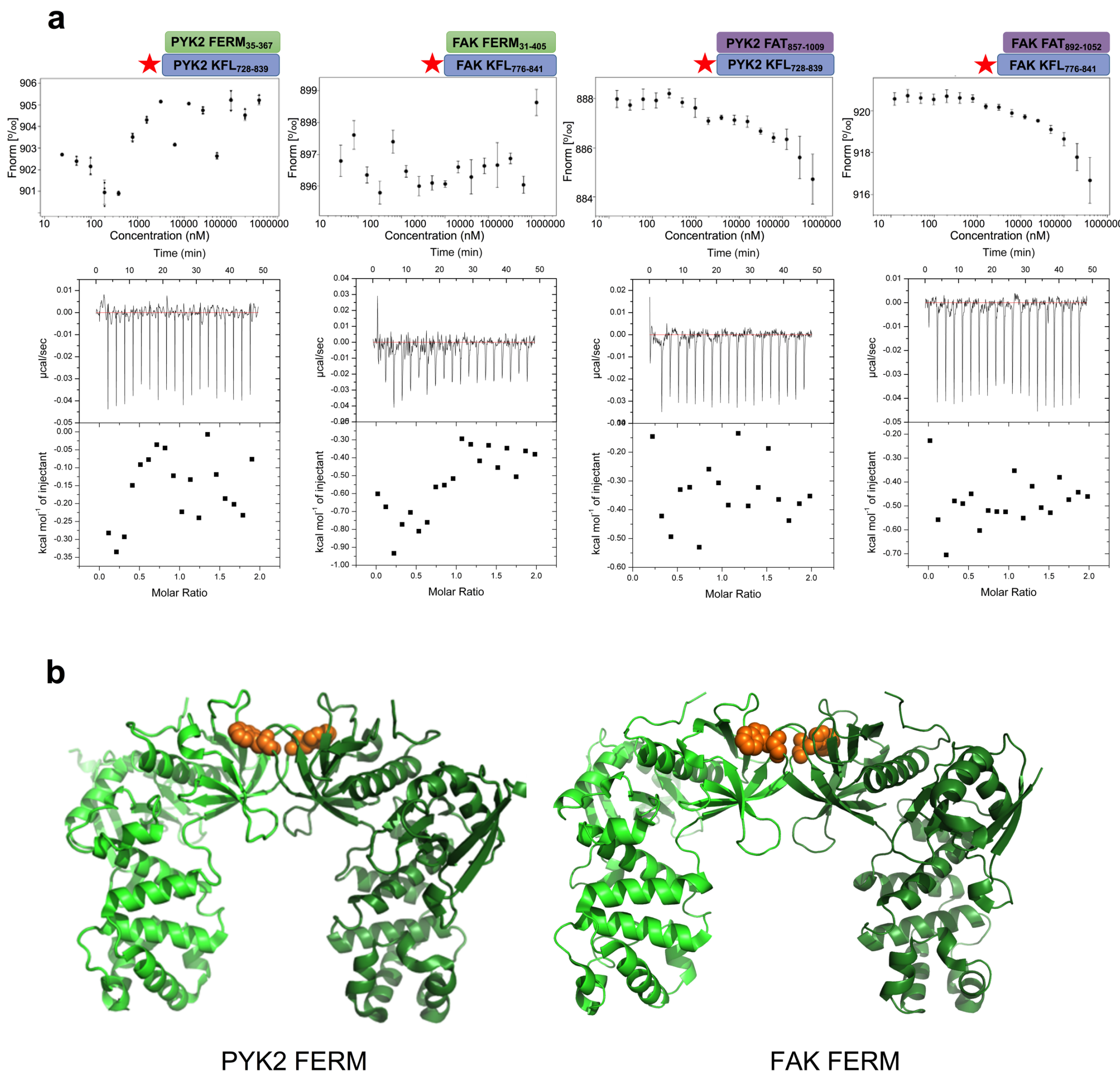

Supplementary Figure 12. Probing intramolecular interactions. (a) MST (*top)* and ITC *(bottom)* analyses to probe binding between the KFL of PYK2 or FAK and their other protein domains. The red star indicates the fluorescently labelled protein. **(b)** The 3D crystal structures of the FERM domain of PYK2 (PDB 4eku) and FAK (PDB 4yn0) contain the same dimers in the crystal lattice (monomers are shown in light and dark green). W266, which is critical for dimerization in FAK FERM, and the corresponding PYK2 W273 are shown as orange sphere models. Binding experiments are represented as (mean ± SD, n=3).

Supplementary Tables

Supplementary Table 1. CD analysis. *Top*: Secondary structure content predicted by sequence analysis PSIPRED[4], and derived from CDdata using the CAPITO web-server (capito.uni-jena.de;[10]) with JASCO ASCII output. *Bottom:* Helical content analysis for PYK2 $KFL_{728–839}$, CaM and PYK2 $KFL_{728–839}$: CaM complex showing no gain in PYK2 $KFL_{728–839}$ helicity upon CaM interaction

Construct	Predicted helical content	Helical content CD spectra		
		α-helix	β-sheets	Random coils
PYK2 $KFL_{728-839}$ – Ca^{2+}	41%	36%	2%	62%
PYK2 $KFL_{728-839}$ – EGTA	41%	36%	1%	63%
CaM - Ca^{2+}	-	65%	2%	33%
CaM - EGTA	-	61%	3%	36%
PYK2 $KFL_{728-839}$: CaM - Ca^{2+}	-	46%	3%	51%
PYK2 $KFL_{728-839}$: CaM - EGTA	-	49%	2%	49%
FAK $KFL_{776-841}$	35%	27%	2%	71%

Construct	No. of residues	No. of helical residues
PYK2 $KFL_{728-839}$ – Ca^{2+}	128	46
CaM - Ca^{2+}	164	107
PYK2 $KFL_{728-839}$: CaM - Ca^{2+}	292	134
PYK2 $KFL_{728-839}$ – EGTA	128	46
CaM - EGTA	164	100
PYK2 $KFL_{728-839}$: CaM - EGTA	292	143

Supplementary Table 2. SAXS analysis

SAXS protein sample	*Mw* calc. (kDa)	Number of amino acids (N)	Globular proteins[11] R_g (Å) = 6.5 * $M^{1/3}$		Unfolded proteins[11] R_g (Å) = 8.05 * $M^{0.522}$		Chemically denatured[12] $R_g = R_0 N^v$		Intrinsically disordered[12] $R_g = R_0 N^v$				
			R_g (monomer) (Å)	R_g (dimer) (Å)	R_g (monomer) (Å)	R_g (dimer) (Å)	R_g (monomer) (Å)	R_g (dimer) (Å)	R_g (monomer) (Å)	R_g (dimer) (Å)	R_g (exp) (Å)	D_{max}	*Mw* SAXS (kDa)
PYK2 $KFL_{728\text{-}839}$	15.03	128	15.76	19.86	32.25	46.31	35.13	53.17	31.97	45.91	30.31	110	41.96
MBP–PYK2 $KFL_{728\text{-}839}$	55.88	505	24.84	31.30	65.75	94.41	79.82	120.82	65.46	93.99	44.88	137	110.55

M = Mw in kDa, N = number of amino acids.
Chemically denatured R_0=1.93 ± 0.076 and v=0.598 ± 0.028.
Intrinsically disordered R_0=2.54 ± 0.01 and v=0.522 ± 0.01.

Supplementary Table 3. Residues involved in PYK2 $KFL_{728\text{-}839}$ dimerization

Residues showing significantly altered backbone amide signals >Interquartile range of CSPs
T728, L735, T749, T751, H769, K775, F784, A799, K803, M804, I807, L808, E817, L822, Q824, Y834, K838

Supplementary Table 4. Summary of residues involved in the CaM-PYK2 KFL association

	$+Ca^{2+}$										+EGTA									
	PYK2 $KFL_{728\text{-}839}$				CaM						PYK2 $KFL_{728\text{-}839}$				CaM					
	All (728-839)	%	Helix (791-812)	%	All (1-148)	%	N-lobe (1-80)	%	C-lobe (81-148)	%	All (728-839)	%	Helix (791-812)	%	All (1-148)	%	N-lobe (1-80)	%	C-lobe (81-148	%
Total residues	112	100	22	19.6	148	100	80	54.1	68	45.9	112	100	22	19.6	148	100	80	54.1	68	45.9
Total Interactions	40	35.7	9	40.9	31	20.9	19	23.8	12	16.1	23	20.5	8	36.4	24	16.2	8	10.0	16	23.5
Hydrophobic	20	50	6	66.7	17	54.9	11	57.9	6	50.0	14	60.9	5	62.5	9	37.5	4	50	5	31.2
Polar	9	22.5	1	11.1	6	19.3	4	21.1	2	16.7	2	8.70	2	25	5	20.8	2	25	3	18.8
Positively charged	5	12.5	1	11.1	2	6.5	2	10.5	0	-	7	30.4	1	12.5	1	4.2	0	-	1	6.2
Negatively charged	6	15	1	11.1	6	19.3	2	10.5	4	33.3	0	-	0	-	9	37.5	2	25	7	43.8
pI binding	5.10	-	6.05	-	4.14	-	6.19	-	3.50	-	11.17	-	8.75	-	3.58	-	3.56	-	3.77	-
pI total	6.64	-	5.01	-	4.09	-	4.07	-	4.12	-	6.64	-	5.02	-	4.09	-	4.02	-	4.12	-
Hydropathicity binding	-0.095	-	0.478	-	0.271	-	0.337	-	0.158	-	0.183	-	3.12	-	0.963	-	-0.450	-	-1.219	-
Hydropathicity total	-0.900	-	-1.372	-	-0.654	-	-0.551	-	-0.772	-	-0.960	-	-1.377	-	-0.654	-	-0.551	-	-0.772	-

Supplementary Table 5. Nuclear magnetic resonance experiments for CaM and PYK2 $KFL_{728–839}$

Study	Transmitter frequency	Experiments	Protein concentration	Solution conditions
PYK2 $KFL_{728–839}$ assignment	950 MHz	^1H-^{15}N HSQC, HNCα, HNCO, HNCαCβ, HN(Cα)CO, HN(CO)Cα, CβCα(CO)NH, and ^1H-^{15}N TROSY	250 µM	20 mM HEPES pH 6.5, 150 mM NaCl, 1 mM TCEP
Ca^{2+}/CaM assignment	700 MHz	^1H-^{15}N HSQC, HNCα, HNCO, HNCαCβ, HN(Cα)CO, HN(CO)Cα, and CβCα(CO)NH	1200 µM	20 mM HEPES pH 6.5, 150 mM NaCl, 1 mM TCEP, 5 mM Ca^{2+}
Apo-CaM assignment	950 MHz	^1H-^{15}N HSQC, HNCα, HNCO, HNCαCβ, HN(Cα)CO, HN(CO)Cα, and CβCα(CO)NH	1200 µM	20 mM HEPES pH 6.5, 150 mM NaCl, 1 mM TCEP, 1 mM EGTA
Assignment confirmation	950 MHz	3D ^1H-^{15}N NOESY	250 µM	20 mM HEPES pH 6.5, 100 mM NaCl, 1 mM TCEP
Ca^{2+}/CaM titrations on PYK2 $KFL_{728–839}$	700 MHz	^1H-^{15}N HSQC	150 µM	20 mM HEPES pH 6.5, 150 mM NaCl, 1 mM TCEP, 5 mM Ca^{2+}
Apo-CaM titrations on PYK2 $KFL_{728–839}$	700 MHz	^1H-^{15}N HSQC	150 µM	20 mM HEPES pH 6.5, 150 mM NaCl, 1 mM TCEP, 1 mM EGTA
PYK2 $KFL_{728–839}$ titrations on Ca^{2+}/CaM	700 MHz	^1H-^{15}N HSQC	150 µM	20 mM HEPES pH 6.5, 150 mM NaCl, 1 mM TCEP, 5 mM Ca^{2+}
PYK2 $KFL_{728–839}$ titrations on apo-CaM	700 MHz	^1H-^{15}N HSQC	150 µM	20 mM HEPES pH 6.5, 150 mM NaCl, 1 mM TCEP, 1 mM EGTA
PYK2 $KFL_{728–839}$ titrations on PYK2 $KFL_{728–839}$	700 Mhz	^1H-^{15}N HSQC	100 µM:10µM	20 mM HEPES pH 6.5, 150 mM NaCl, 1 mM TCEP
PYK2 $KFL_{728–839}$ measured on 950 Mhz and 700 Mhz	950 Mhz and 700 Mhz	^1H-^{15}N HSQC	100 µM	20 mM HEPES pH 6.5, 150 mM NaCl, 1 mM TCEP

CaM, calmodulin; PYK2, protein tyrosine kinase 2-beta; HSQC, heteronuclear single quantum coherence; NOESY, nuclear Overhauser effect spectroscopy

SUPPLEMENTARY REFERENCES

1. Kohno T, Matsuda E, Sasaki H, Sasaki T. Protein-tyrosine kinase CAKbeta/PYK2 is activated by binding Ca2+/calmodulin to FERM F2 alpha2 helix and thus forming its dimer. *The Biochemical journal* **410**, 513-523 (2008).

2. Xie J*, et al.* Analysis of the calcium-dependent regulation of proline-rich tyrosine kinase 2 by gonadotropin-releasing hormone. *Molecular endocrinology* **22**, 2322-2335 (2008).

3. Rost B. PHD: predicting one-dimensional protein structure by profile-based neural networks. *Methods Enzymol* **266**, 525-539 (1996).

4. McGuffin LJ, Bryson K, Jones DT. The PSIPRED protein structure prediction server. *Bioinformatics* **16**, 404-405 (2000).

5. Cuff JA, Clamp ME, Siddiqui AS, Finlay M, Barton GJ. JPred: a consensus secondary structure prediction server. *Bioinformatics* **14**, 892-893 (1998).

6. Lupas A, Van Dyke M, Stock J. Predicting coiled coils from protein sequences. *Science* **252**, 1162-1164 (1991).

7. Simm D, Hatje K, Kollmar M. Waggawagga: comparative visualization of coiled-coil predictions and detection of stable single alpha-helices (SAH domains). *Bioinformatics* **31**, 767-769 (2015).

8. Mruk K, Farley BM, Ritacco AW, Kobertz WR. Calmodulation meta-analysis: predicting calmodulin binding via canonical motif clustering. *J Gen Physiol* **144**, 105-114 (2014).

9. Shen Y, Bax A. Protein backbone and sidechain torsion angles predicted from NMR chemical shifts using artificial neural networks. *J Biomol NMR* **56**, 227-241 (2013).

10. Wiedemann C, Bellstedt P, Gorlach M. CAPITO--a web server-based analysis and plotting tool for circular dichroism data. *Bioinformatics* **29**, 1750-1757 (2013).

11. Bernado P, Blackledge M. A self-consistent description of the conformational behavior of chemically denatured proteins from NMR and small angle scattering. *Biophys J* **97**, 2839-2845 (2009).

12. Bernado P, Svergun DI. Structural analysis of intrinsically disordered proteins by small-angle X-ray scattering. *Mol Biosyst* **8**, 151-167 (2012).
