# Supplementary material for: PYK2 senses calcium through a disordered dimerization and calmodulin-binding element
Source: Commun Biol. 2022 Aug 9;5:800. doi: 10.1038/s42003-022-03760-8 (PMC9363500; doi:10.1038/s42003-022-03760-8)
Supplement: Supplementary file 3 — Description of Additional Supplementary Files [file 42003_2022_3760_MOESM3_ESM.pdf]

Description of Additional Supplementary Files

File name: Supplementary Data
Description: The source data underlying graphs, plots, and charts in the manuscript.
